# Clinico-Hematological Profile of Acute Myeloid Leukemia: Experience From a Tertiary Care Cancer Center in North India

**DOI:** 10.7759/cureus.50869

**Published:** 2023-12-20

**Authors:** Arushi Vemprala, Smeeta Gajendra, Ritu Gupta, Deepshi Thakral, Sameer Bakhshi, Ranjit K Sahoo, Rachna Seth, Ashish Datt Upadhyay

**Affiliations:** 1 Department of Laboratory Oncology, Dr. B. R. Ambedkar Institute Rotary Cancer Hospital, All India Institute of Medical Sciences, New Delhi, IND; 2 Department of Medical Oncology, All India Institute of Medical Sciences, New Delhi, IND; 3 Division of Pediatric Oncology, Department of Pediatrics, All India Institute of Medical Sciences, New Delhi, IND; 4 Clinical Research Unit, All India Institute of Medical Sciences, New Delhi, IND

**Keywords:** acute myeloid leukemia, aml-eto, flt3, npm1, flow cytometric immunophenotyping, aml

## Abstract

Introduction: Complete diagnosis of acute myeloid leukemia (AML) requires knowledge of clinical information combined with morphologic evaluation, immunophenotyping, karyotyping, and molecular genetic testing. The study intends to evaluate the demographic profile, clinical workup, and investigation, including flow cytometric immunophenotyping, in adult and pediatric age groups of AML.

Materials and methods: This is a retrospective study of AML patients treated between January 2017 and December 2021. Clinical and demographic characteristics and investigation findings were recorded from case files and the hematology database.

Result: A total of 896 cases of AML were registered during the given period, of which 819 cases were de-novo AML. Among those 819 cases, more than two-thirds of cases, i.e., 78.9% (N = 646), received induction chemotherapy. A significantly higher male-to-female ratio was observed (1.5:1). The median age was 22 years. The median time for diagnosis was three days and the median time for treatment intervention was four days. There were significant differences in the Eastern Cooperative Oncology Group (ECOG) performance status scores between pediatric and adult AML patients. Pediatric AML patients presented with better ECOG performance scores (ECOG performance scores 0 and 1) than adult patients (74.76% vs. 43.14%, p < 0.001). Further comparing adult vs. pediatric AML patients, normal karyotype (60.56% vs. 31.93%, p < 0.001) and NPM1 (22.25% vs. 6.72%, p < 0.001) and FLT3-ITD mutations (20.28% vs. 7.98%, p<0.001) were more common in the adult group, whereas AML-ETO (40.76% vs. 16.34%, p < 0.001) was more common in the pediatric group.

Conclusion: The study highlights the presenting age is lower than global figures. The median time for initial diagnosis and the start of treatment is within the acceptable norms. Normal karyotype and NPM1 and FLT3 mutations were common in adult AML patients, whereas AML-ETO was more common in the pediatric cohort. These findings will help plan prospective studies and see the correlation with treatment outcomes. The laboratory workup practice currently complies with the standard guidelines at our center.

## Introduction

Acute myeloid leukemia (AML) is a clonal expansion of myeloid blasts in the peripheral blood, bone marrow, and other tissues. It is a heterogeneous disease due to variations in genetic aberrations and immunophenotypes among patients and between malignant subclones coexisting within a single patient. AML accounts for 15-20% of all cases of acute leukemia in children below 15 years; however, it is the most common type accounting for 80-90% of cases of acute leukemia in adults. Worldwide, the annual incidence is approximately 2.5-3 cases per 100,000 population per year [1]. The majority present with symptoms related to anemia, infection, and bleeding with or without focal neurological symptoms, but a few are asymptomatic and are diagnosed only with laboratory abnormalities. It can occur at all ages but pediatric AML is a rare disease, accounting for 15-20% of childhood acute leukemia with a median age of six years to 10.5 years, as reported in the literature [2,3]. The incidence increases with age. AML is the most common acute leukemia in adults and accounts for approximately 80% of cases in this age group with a median age of diagnosis of 68 years [4].

In AML, age is the most prominent patient-specific risk factor, while chromosomal aberrations are the strongest disease-specific risk factors [5]. Diagnostic laboratory workup and time to diagnosis are important aspects of outcome, and to the best of our knowledge, there are no Indian data highlighting this issue.

The current study intends to evaluate demographic profile and clinical and laboratory investigations, including morphology, flowcytometric immunophenotyping, cytogenetics, and molecular workup, along with risk stratification in adult and pediatric age groups at a tertiary care cancer center in India. The findings will be relevant for planning management for many low and middle-income countries with limited resources for diagnosis and treatment.

## Materials and methods

Patients

**Figure 1:**
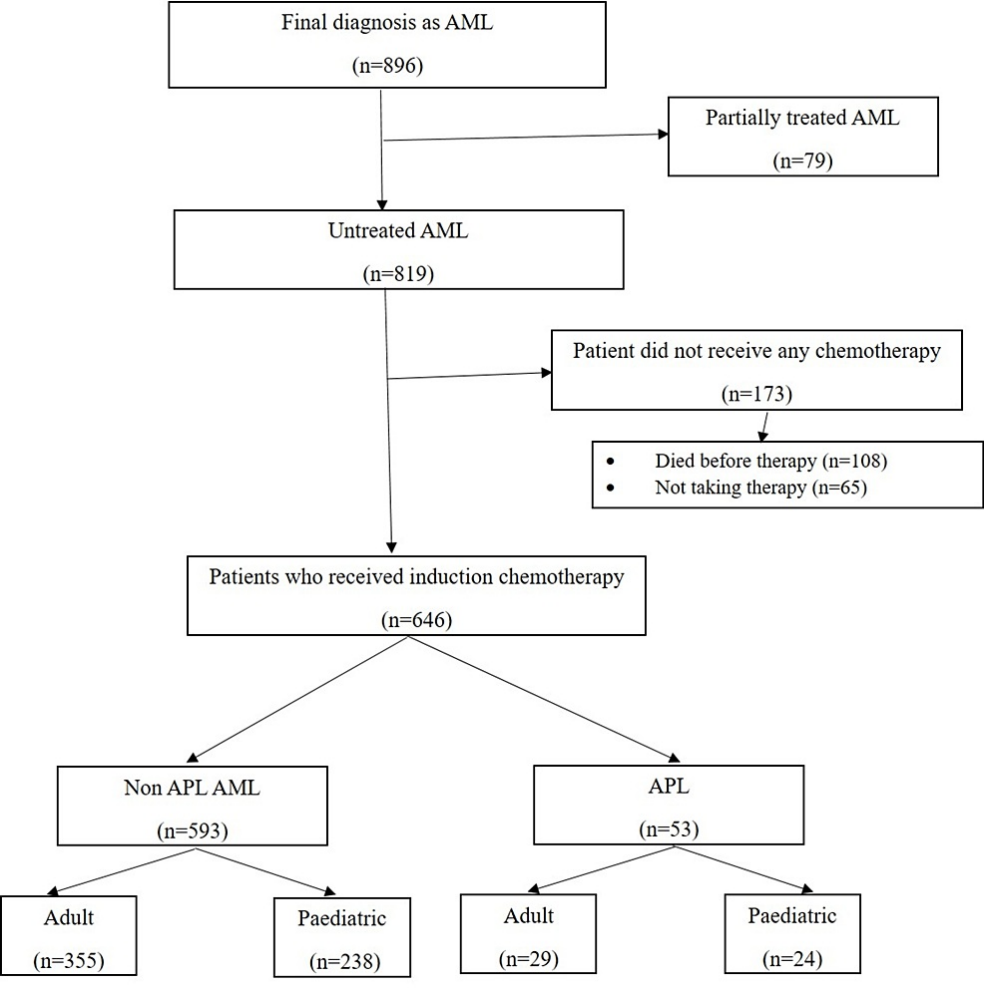
Consort diagram of acute myeloid leukemia patients between January 2017 and December 2021 AML: acute myeloid leukemia; APL: acute promyelocytic leukemia.

A retrospective analysis was conducted from January 2017 to December 2021 using case records of the suspected cases of AML at our center. File records and computer data available in the hospital information system of patients were evaluated. Diagnosis of AML was made based on the morphology, cytochemistry, immunophenotype, cytogenetics, and/or molecular features as per the World Health Organization guidelines [1]. Detailed demography and clinical data, including presenting symptoms, family and personal history, any prior treatment, physical examination findings, etc., were recorded. Relevant investigations, including complete blood count (CBC), lactate dehydrogenase (LDH), and other important laboratory tests, as well as radiological findings, were also recorded. Patients with final diagnoses other than AML, patients with AML who were partially treated outside, and those who did not receive any chemotherapy were excluded (Figure 1). Figure 1 shows the distribution of AML cases.

Morphological assessment

Peripheral blood and bone marrow smears were stained with Jenner-Giemsa stain. Detailed morphological evaluations, including differential count, blast percentage, and blast morphology, were independently evaluated by two hematopathologists. Two hundred leukocytes on blood smears and 500 nucleated cells on bone marrow smears were counted for differential percentages. Cytochemical stains like myeloperoxidase (MPO) and nonspecific esterase (NSE) were performed in all cases.

Flow cytometric immunophenotyping

Peripheral blood or bone marrow sample at diagnosis was analyzed for flow cytometric immunophenotyping (FCMI). All the samples were collected in ethylenediaminetetraacetic acid (EDTA) and processed within 24 hours of collection with bulk-lysis and stain protocol used at our center [6]. FCMI was done using three lasers and a 10-color Gallios flow cytometer (Beckman Coulter, La Brea, CA). The panel of antibodies for diagnosis of AML included CD45, CD34, human leukocyte antigen (HLA)-DR, cMPO, cCD79a, cCD3, sCD3, CD13, CD33, CD117, CD123, CD64, CD14, CD11b, CD11c, CD16, CD36, CD56, CD38, CD19, CD7, and CD4. Additional markers such as CD41, CD42, and CD61 in suspected cases of acute megakaryoblastic leukemia, CD2, CD9, CD15, CD18, and CD25 in suspected cases of acute promyelocytic leukemia (APL), and CD71 and CD235a in cases of pure erythroid leukemia were analyzed. A total of 100,000 events were acquired at diagnosis. Data analysis was done using Kaluza software version 2.0 (London, UK). The cut-off of 20% or more of leukemic cells expressing the marker was applied to determine the positive expression of an antigen, whereas, for selected markers (e.g., cytoplasmic CD3, MPO, TdT, CD34, and CD117), a lower cut-off of 10% was applied [7].

Molecular genetic studies

Conventional karyotyping was done using Wright-Giemsa staining. For detection of balanced translocations, reverse transcription-polymerase chain reaction (RT-PCR) was performed with complementary DNA (cDNA) prepared from RNA isolated using a commercially available cDNA synthesis kit. Translocations tested by RT-PCR were t(8;21)(q22;q22), RUNX1-RUNX1T1, inv(16)(p13.1q22) or t(16;16)(p13.1;q22), CBFB-MYH11 and t(15;17)(q22;q12), and PML-RARA. Mutations including tetranucleotide insertion in exon 12 of NPM1-A and FLT3-ITD were detected by fragment analysis in a multiplex assay. FLT3-TKD (D835Y) mutation was detected by allele-specific oligonucleotide (ASO)-polymerase chain reaction (PCR).

Risk stratification

Risk stratification was done as per the European LeukemiaNet (ELN) 2022 guidelines and patients were categorized into favorable, intermediate, and unfavorable risk sub-groups [8]. Induction chemotherapy included standard induction therapy, which was given according to the clinical judgment of the treating physician. Induction therapy was chosen based on the fitness of the patients (age and performance status), the resources of patients, and the hospital. The majority of the patients received “7+3” induction chemotherapy with daunorubicin (60 mg/m2, day one to day three) and cytarabine (100 mg/m2/day continuous infusion, day one to day seven). The majority of older patients received low-intensity induction (hypomethylating agent, low-dose Ara-C with or without venetoclax). A few patients also received other chemotherapy regimens like 2+5, MRC-15, MRC-12, and ADE (cytarabine, daunorubicin, and etoposide).

Statistical analysis

All the statistical data analysis was analyzed by Stata version 14 (StataCorp, College Station, TX) and presented in median (Min-Max) and frequency (%). Categorical variables were compared by chi-square or Fisher's exact test. Continuous variables were compared by independent t-test (following a normal distribution) or Wilcoxon rank sum test (non-normal distributed variables). A p-value less than 0.05 was considered statistically significant.

## Results

A total of 896 cases were diagnosed as AML during the study period, of which 79 cases were partially treated prior to reporting at our center. A total of 646 cases of de novo AML were analyzed for demographic details, laboratory parameters, and risk stratification.

Only one-third of the patients (N = 250; 38.7%) belong to Delhi/NCR (National Capital Region) and the remaining are from other northern states of India. The age ranged from one month to 79 years, with a median age of 22 years. The male-to-female ratio was 1.5:1. Among 646 cases, 53 cases were diagnosed as APL, and 593 cases with non-APL AML (Figure 1). Of 593 non-APL AML cases, 355 were adult and 238 were pediatric (age < 18 years) AML cases. The median time for diagnosis was three days (from the date of registration to the date of diagnosis). The median time taken from diagnosis to start chemotherapy was four days (from the date of diagnosis to the date of starting induction chemotherapy).

Among pediatric non-APL AML cases (N = 238), the median age of presentation was eight years. The median duration of illness prior to diagnosis was 1.5 months (range: one week to 12 months). Fever (N = 203; 85.29%) was the most common presenting symptom followed by fatigue (N = 95; 39.91%) and bleeding (N = 75; 31.51%). Pallor (N = 204; 85.29%) was the most common sign followed by lymphadenopathy (N = 131; 55.04%) and hepatomegaly (N = 91; 38.24%). A total of 211/238 (88.66%) cases presented with anemia (median hemoglobin: 7.3 g/dl; range: 1.7-16.4 g/dl). Leucopenia and leucocytosis were observed in 20/238 (8.40%) and 161/238 (67.65%) of cases, respectively (median total leucocyte count (TLC): 24.03x10^9^/L; range: 0.23-811.9x109/L), with the presence of hyperleucocytosis in 38/238 (15.97%) of cases. Thrombocytopenia with platelet counts <100x10^9^/L was observed in 193/238 (81.09%) cases, whereas one of 238 (0.42%) patients showed thrombocytosis (median platelet count: 35x109/L; range: 5-898x109/L). Increased LDH level was seen in 225/238 (94.54%) cases (median LDH: 515 U/L; range: 154-8930 U/L). On morphological examination, the percentage of blasts ranged from 0% to 99% (median: 51%) on peripheral blood and 18% to 100% (median: 65%) on bone marrow. Conventional karyotyping revealed a normal karyotype in 76/238 (31.93%), and 162/238 (68.07%) cases showed cytogenetic abnormalities. On molecular evaluation, 16/238 (6.72%) were NPM1 positive, 19/238 (7.98%) were FLT3-ITD positive and 9/238 (3.78%) were FLT3-TKD positive. Double mutations of NPM1 and FLT3-ITD were seen in four (1.68%) pediatric AML patients. AML-ETO was positive in 97/238 (40.76%) and CBFB-MYH11 was positive in 12/238 (5.04%) cases of pediatric AML. ELN-molecular risk stratification showed 123/238 (51.68%) patients in ELN favorable risk category whereas ELN intermediate and adverse risk groups comprised 103/238 (43.28%) and 12/238 (5.05%) patients, respectively.

Among adult non-APL AML cases (N = 355), the median age of presentation was 39 years. The median duration of illness prior to diagnosis was 1.5 months (range: one week to 24 months). Fever (N = 270; 76.06%) was the most common symptom followed by fatigue (N = 225; 63.42%) and bleeding (N = 99; 28.02%). Pallor (N = 299; 84.4%) was the most common sign followed by lymphadenopathy (N = 106; 29.84%) and hepatomegaly (N = 82; 23.17%). Around 99/238 (27.89%) patients had co-morbidity, diabetes mellitus being the most common, followed by hypertension and hypothyroidism. A total of 296/355 (83.38%) cases had anemia (median hemoglobin: 7.4g/dl; range: 1.9-16.0g/dl). Leucopenia and leucocytosis were observed in 67.355 (18.87%) and 201/355 (56.62%) of cases, respectively (median TLC: 13.73x10^9^/L; range: 0.5-984.9x10^9^/L), with the presence of hyperleucocytosis in 43/355 (12.11%) of cases. Thrombocytopenia and thrombocytosis were observed in 281/355 (79.15%) and 3/355 (0.85%) cases, respectively (median platelet count: 40x10^9^/L; range: 1-657x10^9^/L). Increased LDH level was seen in 301/355 (84.79%) cases. On morphological examination, the percentage of blasts ranged from 0% to 98% (median: 55%) on peripheral blood and 4% to 100% (median: 75%) on bone marrow. Conventional karyotyping revealed a normal karyotype in 215/355 (60.56%) cases and cytogenetic abnormalities were seen in 140/355 (39.44%) cases of adult AML. On molecular evaluation, 79/355 (22.25%) were NPM1 positive, 72/355 (20.28%) were FLT3-ITD positive, and 24/355 (6.76%) were FLT3-TKD positive. Double mutations of NPM1 and FLT3-ITD were seen in 36 (10.14%) adult AML patients. AML-ETO was positive in 58/355 (16.34%) and CBFB-MYH11 in 13/355 (3.66%) cases. About 114/355 (32.11%) patients belonged to ELN favorable risk category whereas 229/355 (64.51%) and 12/355 (3.38%) patients belonged to intermediate and adverse risk groups, respectively.

A comparison of different clinicopathological parameters between adult and pediatric groups of patients is demonstrated in Table 1.

**Table 1:** Demography and clinicopathological profile of the acute myeloid leukemia (non-acute promyelocytic leukemia) study cohort AML: acute myeloid leukemia; APL: acute promyelocytic leukemia; N: number of patients; BSA: body surface area; BMI: body mass index; NA: not applicable; ECOG: Eastern Cooperative Oncology Group; H/O/: history of; CNS: central nervous system; Hb: hemoglobin; TLC: total leucocyte count; PS: peripheral smear; BM: bone marrow; MRC: myelodysplasia-related changes; NOS: not otherwise specified; DS: down syndrome; LDH: lactate dehydrogenase; WHO: World Health Organization; ELN: European LeukemiaNet; FAB: French-American-British.

Patient characteristics	All cases (N = 593)	Pediatric (N = 238)		Adult (N = 355)	p-value
Median age (years)	22	8		39	NA
Male, N (%)	356 (60.03%)	155 (65.13%)		201 (56.62%)	0.038
Female: N (%)	237 (39.97)	83 (34.87%)		154 (43.38%)	
M:F ratio	1.5:1	1.9:1		1.3:1	
Median BSA (m^2^) (range)	1.48 (0.17-2.52)	0.85 (0.17-1.82)		1.60 (1.04-2.52)	NA
Median BMI (range)	19.95 (8.9-51.1)	14.7 (8.9-27.0)		22.3 (12.8-51.1)	0.352
ECOG performance score					
ECOG 0-1	305/556 (54.86%)	154/206 (74.76%)		151/350 (43.14%)	<0.001
ECOG 2-4	251/556 (45.14%)	52/206 (25.24%)		199/350 (56.86%)	
Fever, N (%)	473 (79.76%)	203 (85.29%)		270 (76.06%)	0.032
Fatigue, N (%)	320 (53.91%)	95 (39.91%)		225 (63.42%)	<0.001
Bleeding, N (%)	174 (29.34%)	75 (31.51%)		99 (27.89%)	0.459
Pallor, N (%)	503 (84.82%)	204 (85.29%)		299 (84.23%)	0.678
Lymphadenopathy, N (%)	237 (39.97%)	131 (55.04%)		106 (29.86%)	<0.001
Hepatomegaly, N (%)	173 (29.17%)	91 (38.24%)		82 (23.10%)	0.001
Splenomegaly, N (%)	138 (23.27%)	65 (27.31%)		73 (20.56%)	0.120
Gum hypertrophy, N (%)	78 (13.15%)	26 (10.96%)		52 (14.65%)	0.285
Presence of co-morbidities, N (%)	99 (16.69%)	0 (0.00%)		99 (27.89%)	<0.001
H/O previous malignancy, N (%)	33 (5.56%)	7 (2.94%)		26 (7.32%)	0.071
CNS disease at diagnosis, N (%)	11 (1.85%)	8 (3.36%)		3 (0.85%)	0.048
Extra-medullary disease, N (%)	19 (3.20%)	11 (4.62%)		8 (2.25%)	0.176
Median Hb, g/dl (range)	7.4 (1.7-16.4)	7.3 (1.7-16.4)		7.4 (1.9-16.0)	0.201
Median TLC: x10^9^/L (range)	19.75 (0.23-984.9)	24.03 (0.23-811.9)		13.73 (0.9-984.9)	0.001
Hyperleucocytosis (TLC > 100 x10^9^/L), N (%)	81 (13.66%)	38 (15.97%)		43 (12.11%)	0.189
Median platelets: x10^9^/L (range)	38 (1-898)	35 (5-898)		40 (1-657)	0.163
Median LDH (range): U/L (range)	493 (102-8930)	515 (154-8930)		456 (102-8885)	0.393
Median PS blast% (range)	52 (0-99)	51 (0-99)		55 (0-98)	0.770
Median BM blast% (range)	70 (0-100)	65 (18-100)		75 (4-100)	0.019
FAB subtype					
M0, N (%)	19 (3.20%)	9 (3.78%)		10 (2.82%)	0.002
M1, N (%)	81 (13.66%)	33 (13.87%)		48 (13.52%)	
M2, N (%)	344 (58.01%)	141 (59.24%)		203 (57.18%)	
M4, N (%)	73 (12.31%)	25 (10.50%)		48 (13.52%)	
M5, N (%)	61 (10.29%)	17 (7.14%)		44 (12.39%)	
M6, N (%)	2 (0.34%)	1 (0.42%)		1 (0.28%)	
M7, N (%)	13 (2.19%)	12 (5.04%)		1 (0.28%)	
Abnormal karyotype, N (%)	302 (50.93%)	162 (68.07)%		140 (39.44%)	<0.001
WHO subtype					
AML with recurrent cytogenetic abnormalities, N (%)	237 (39.97%)		107 (44.96%)	130 (36.62%)	0.003
AML-MRC, N (%)	12 (2.02%)		1 (0.42%)	11 (3.10%)	
Therapy-related AML, N (%)	18 (3.04%)		3 (1.26%)	15 (4.23%)	
AML-NOS, N (%)	306 (51.60%)		114 (47.90%)	192 (54.08%)	
Myeloid sarcoma, N (%)	11 (1.85%)		5 (2.10%)	6 (1.69%)	
AML associated with DS, N (%)	9 (1.52%)		8 (3.36%)	1 (0.28%)	
Molecular testing					
NPM1 mutated, N (%)	95 (16.02%)		16 (6.72%)	79 (22.25%)	<0.001
FLT3-ITD mutated, N (%)	91 (15.35%)		19 (7.98%)	72 (20.28%)	<0.001
Both NPM1+FLT3-ITD mutation	40 (6.74%)		4 (1.68%)	36 (10.14%)	<0.001
FLT3-TKD mutated, N (%)	33 (5.56%)		9 (3.78%)	24 (6.76%)	0.260
AML-ETO positive, N (%)	155 (26.14%)		97 (40.76%)	58 (16.34%)	<0.001
CBFB-MYH11 positive, N (%)	25 (4.21%)		12 (5.04%)	13 (3.66%)	0.445
ELN risk group					
Favorable, N (%)	39.84%		123 (51.68%)	114 (32.11%)	<0.001
Intermediate, N (%)	56.21%		103 (43.28%)	229 (64.51%)	
Adverse, N (%)	3.95%		12 (5.04%)	12 (3.38%)	

There was a higher male preponderance in pediatric than in adult patients (p = 0.03). Lymphadenopathy, hepatomegaly, and CSF involvement at diagnosis were also more common in pediatric than adult AML cases. Pediatric AML cases displayed higher median TLC (24.03 vs. 13.73 x109/L, p = 0.001) and lower bone marrow blast % (65 vs. 75%, p 0.019) than adult AMLs. Normal karyotype, NPM1 mutation, FLT3 mutations, and double mutation (NPM1 and FLT3) were more common in the adult group, whereas AML-ETO was more common in the pediatric group (p < 0.001).

Among APL cases (N = 53), there were 24 pediatric and 29 adult cases. The male-to-female ratio was 1.65:1. The median age of presentation was 22 years (range: 1-65 years). The median duration of illness prior to diagnosis was 0.5 months (range: one week to four months). Fever (N = 43; 81.13%) was the most common symptom followed by bleeding (N = 37; 69.81%) and fatigue (N = 32; 60.38%). Pallor (N = 51; 96.22%) was the most common sign followed by hepatomegaly (N = 16; 30.19%) and lymphadenopathy (N = 11; 20.75%). Only 5/53 (9.43%) patients had co-morbidity. No patients had a prior history of malignancy or extra-medullary disease at diagnosis. One patient (1.89%) had CNS involvement at diagnosis. A total of 46/53 (86.79%) cases had anemia (median hemoglobin: 7.2 g/dl; range: 3.6-13.6 g/dl). Leucopenia and leucocytosis were observed in 17/53 (32.08%) and 25/53 (47.17%) of cases, respectively (median TLC: 10.9x10^9^/L; range: 0.58-178.57x109/L), with the presence of hyperleucocytosis in 4/53 (7.55%) cases. Thrombocytopenia was observed in 49/53 (92.45%) cases (median platelet count: 25x10^9^/L; range: 5-215x10^9^/L). Increased LDH level was seen in 42/53 (79.25%) cases with a median LDH value of 418 U/L (range: 196-1698 U/L). On morphological examination, the percentage of blasts+ abnormal promyelocytes ranged from 2% to 100% (median: 75%) on peripheral blood and 16% to 100% (median: 90%) on bone marrow. Conventional risk stratification based on a combination of the white cell count and platelet count at presentation showed 20.75% (N = 11) patients with good risk, 32.08% (N = 17) at intermediate risk, and 47.17% (N = 25) in high-risk categories.

## Discussion

The present study assessed a total of 896 cases of AML, including 646 patients of de novo AML cases evaluated with laboratory investigations, as per institutional protocol. The study criteria were to evaluate the demographic profile and laboratory practice at a tertiary care hospital in India and to see if it complies with the set WHO guidelines. Out of the total cases, only one-third of the patients belonged to Delhi/NCR, and the remaining were from other states of India. The observation highlights the lack of adequate infrastructure in diagnosis and treatment in the neighboring states of Delhi in northern India.

In our study, the median age of presentation was eight years in the non-APL AML pediatric age group compared to 39 years in the adult group and 22 years in the APL patients; however, there is a trend of higher incidence with increasing age in the western literature (median age of 65 years). It was reported that nearly 75% of AML patients were aged 65 years or older in the USA [9]. However, this younger median age is consistent with previous reports from our country and the Asian subcontinent [10,11]. In our study, only 3.9% of patients in our study group were aged 65 years or older. The exact cause is not known, but population demographics and inadequate medical access or care in the elderly age group in our country may be a possible reason for a lower incidence of the disease in the elderly.

The mean time to reach the center for complete evaluation, diagnosis, and treatment after the onset of symptoms was 1.5 months, revealing a health system hurdle. A similar observation was made by several studies emphasizing time lag at health system levels to establish diagnosis and treatment [12,13]. The average time to start treatment in our study was four days. Reports have indicated that treatment outcome is poor when the time to start treatment is more than five days [14]. Another study suggested that it was not related to survival and that it is a feasible approach to wait for laboratory test results including molecular genetic reports so that clinically stable patients are assigned the best available treatment [15]. The waiting time to start treatment in our study is within the acceptable norms.

Symptoms are results of bone marrow failure, tissue infiltration by leukemic cells, or may be due to circulating leukemic cells. Patients usually present with fever (up to 80% of patients), fatigue, abnormal bleeding, weight loss, and body/bone pain. In our study, fever was the most common symptom seen in about 80% of cases, followed by fatigue and bleeding. Such observation was comparable to other studies [10,16]. On physical examination, pallor and features of thrombocytopenia like epistaxis, gingival bleeding, petechial, and ecchymotic patches may be present. Patients may have hepatosplenomegaly, lymphadenopathy, and gingival hypertrophy (usually in AML with monocytic differentiation). In our study, pallor was the most common sign seen in more than 80% of patients, followed by lymphadenopathy and hepatomegaly. The central nervous system involvement in patients with AML is rare, and more common in pediatric patients than in adults [17]. Patients may have headaches, vomiting, and blurring of vision due to CNS involvement. In our study, CNS involvement in AML was 3.36% (N = 8) and 0.85% (N = 3) at the time of diagnosis in pediatric and adult patients, respectively. This lower incidence of CNS involvement may be due to less screening of cerebrospinal fluid at diagnosis. Extra-medullary disease is reported in 2.5%-9.1% of patients with AML and occurs concomitantly, following, or, rarely, antedating the onset of bone marrow leukemia [18]. In our study, extra-medullary disease was observed in 4.62% (N = 11) and 2.25% (N = 8) of pediatric and adult patients, respectively. Conventional cytogenetics analysis is a mandatory component in the diagnostic evaluation of a patient with suspected acute leukemia. Cytogenetic analysis reveals clonal chromosome abnormalities in 70-85% of children with AML, which was similar to our study with 68.07% of pediatric cases with cytogenetic abnormalities [19]. Chromosome abnormalities are detected in approximately 55% of adult AML [20]. In our study, we found 39.44% of cases of adults associated with cytogenetic abnormalities. NPM1 mutations are the most frequent mutation in AML, occurring in 25%-30% of AML patients [21]. FLT3 gene has been found in 20% of all AML cases [22]. In our study, we found overall 16.02% (N = 95/593) and 20.91% (N = 124/593) of patients with NPM1 and FLT3 mutations. In adults, both these mutations, NPM1 and FLT3 (both ITD & TKD), were more common (22.25% and 27.04%, respectively) than in pediatric patients (Table 1; p <0.001), similar to other studies [23,24]. Double mutations of NPM1 and FLT3-ITD were more common in adults than in pediatric AML cases (1.68% vs. 10.14%, <0.001).

On ELN risk stratification, more than half of the pediatric study cohort (N = 123/238; 51.68%) belonged to a favorable risk group, whereas in adults, the intermediate risk group was higher (N = 229/355; 64.51%).

Our study had a few limitations, such as it was a single-center and retrospective study based on hospital and laboratory records. Correlation with treatment history and long-term disease outcome as relapse rate and disease-free survival was not done.

## Conclusions

The current study from a tertiary cancer center highlights that more than 60% of the patients presented from neighboring states, reflecting poor infrastructure and access to inadequate diagnostic and treatment facilities. The mean time to reach the center for complete evaluation, diagnosis, and treatment after the onset of symptoms was 1.5 months, revealing a health system hurdle. Therefore, policymakers should rationally allocate public health resources for early diagnosis and treatment at primary care centers to relieve the early mortality burden of AML and to provide more affordable healthcare facilities. The presenting age in the pediatric group is similar to previous reports, but in adults, it is lower than global figures, perhaps undermining the healthcare needs of the geriatric population. The waiting time to start treatment is within the acceptable norms considering the average time to get laboratory test reports. Normal karyotype, NPM1 mutation, FLT3 mutations, and double mutations (NPM1 with FLT3) were more common in the adult group, whereas AML-ETO was more common in the pediatric group. There is the inclusion of a higher favorable risk group with the absence of co-morbidities in the pediatric group, which may be correlated with better survival in this group, as suggested in the literature.
